# Heat shock proteins and small nucleolar RNAs are dysregulated in a Drosophila model for feline hypertrophic cardiomyopathy

**DOI:** 10.1093/g3journal/jkaa014

**Published:** 2020-11-27

**Authors:** Christian A Tallo, Laura H Duncan, Akihiko H Yamamoto, Joshua D Slaydon, Gunjan H Arya, Lavanya Turlapati, Trudy F C Mackay, Mary A Carbone

**Affiliations:** 1 Department of Biological Sciences, North Carolina State University, Raleigh, NC 27695-7614, USA; 2 The Department of Entomology and Plant Pathology, North Carolina State University, Raleigh, NC 27695-7613, USA; 3 The Center for Human Genetics and Department of Genetics and Biochemistry, Clemson University, Greenwood, SC 29646, USA; 4 The Comparative Medicine Institute, North Carolina State University, Raleigh, NC 27695, USA; 5 The Center for Integrated Fungal Research and Department of Plant and Microbial Biology, North Carolina State University, Raleigh NC 27695-7244, USA

**Keywords:** MYBPC3, cMyBP-C, *Drosophila melanogaster*, feline HCM, hypertrophic cardiomyopathy

## Abstract

In cats, mutations in myosin binding protein C (encoded by the *MYBPC3* gene) have been associated with hypertrophic cardiomyopathy (HCM). However, the molecular mechanisms linking these mutations to HCM remain unknown. Here, we establish *Drosophila melanogaster* as a model to understand this connection by generating flies harboring *MYBPC3* missense mutations (A31P and R820W) associated with feline HCM. The A31P and R820W flies displayed cardiovascular defects in their heart rates and exercise endurance. We used RNA-seq to determine which processes are misregulated in the presence of mutant *MYBPC3* alleles. Transcriptome analysis revealed significant downregulation of genes encoding small nucleolar RNA (snoRNAs) in exercised female flies harboring the mutant alleles compared to flies that harbor the wild-type allele. Other processes that were affected included the unfolded protein response and immune/defense responses. These data show that mutant *MYBPC3* proteins have widespread effects on the transcriptome of co-regulated genes. Transcriptionally differentially expressed genes are also candidate genes for future evaluation as genetic modifiers of HCM as well as candidate genes for genotype by exercise environment interaction effects on the manifestation of HCM; in cats as well as humans.

## Introduction

Cardiovascular disease is the leading cause of death worldwide, accounting for 30% of all deaths. The American Heart Association estimates that over 85 million people in the United States have cardiovascular disease, with an associated healthcare cost of ∼320 billion dollars. Cardiomyopathy refers to disease of the heart muscle and can be classified as either primary or secondary. Secondary cardiomyopathy results from extrinsic factors such as hypertension, ischemia, and metabolic disorders, while primary cardiomyopathy occurs in the absence of extrinsic factors. Hypertrophic cardiomyopathy (HCM) is a primary myocardial disease characterized by thickening of the left ventricle myocardium, impaired diastolic function, myocardial fibrosis, and altered calcium kinetics in the cell (Houston and Stevens 2014; Fraiche and Wang 2016; Kimura 2016). It affects over 1 in 500 people in the general population and ∼700,000–725,000 are affected in the United States (Maron *et al.* 2016; Sen-Chowdhry *et al.* 2016). Patients diagnosed with HCM may experience one or more of the following symptoms: shortness of breath, chest pain during exercise, fainting, heart-palpitations, heart failure, or sudden cardiac death.

In most cases, HCM is caused by mutations in genes encoding proteins involved with sarcomere structure or function and is often inherited as a Mendelian autosomal dominant trait (Bonne *et al.* 1998). Seventy percent of HCM cases have been linked to mutations in 11 sarcomeric genes, leaving ∼30% of cases with unidentified causes (Efthimiadis *et al.* 2014; Roma-Rodrigues and Fernandes 2014). Most mutations associated with HCM occur in the *MYBPC3* gene that encodes myosin binding protein C (cMyBP-C). These are either frame-shift or splice-site mutations resulting in a truncated protein, or missense mutations in which a single DNA nucleotide change results in an amino acid substitution (ClinVar database; https://www.ncbi.nlm.nih.gov/clinvar/) (Landrum *et al.* 2016). As of January 27, 2020, the ClinVar database has curated 1594 *MYBPC3* variants, of which 342 have been classified as pathogenic for HCM. Phenotypic heterogeneity is prevalent within and between families, suggesting that mutations of the sarcomere are not the sole determinant of the HCM phenotype.

The human *MYBPC3* gene encodes the 1274 amino-acid (140 kDa) myosin-associated protein cMyBP-C that contributes to the structural integrity of the sarcomere and regulates myocardial contraction (Hartzell 1985; Gilbert *et al.* 1996; Weisberg and Winegrad 1996). The cMyBP-C protein consists of 11 domains (C0–C10)—8 immunoglobulin-like C2 domains and 3 fibronectin type III domains (Sequeira *et al.* 2014). Five phosphorylation sites have been identified, four are located in the cMyBP-C regulatory region (M-domain) and one is in the proline-alanine rich domain (Gruen *et al.* 1999; Kuster *et al.* 2013). Myocardial contraction is achieved by the sliding movement of myosin-containing thick filaments and the actin-containing thin filaments in the sarcomere. Each cMyBP-C molecule is tethered to the myosin thick filament through its C-terminus (C7–C10) and interacts with actin via its N-terminal domains (C0–C2). The contraction and relaxation processes are triggered by a structural change in the myosin head domains while they are bound to actin and coupled with ATP hydrolysis (Inchingolo *et al.* 2019). In addition to actin and myosin, the sarcomere contains multiple other proteins (including calmodulin, titin, and troponin) that serve as binding partners with cMyBP-C to regulate cardiovascular health (Heling *et al.* 2020).

HCM is a leading cause of cardiovascular sudden death among competitive US athletes (Maron *et al.* 2009, 2016). Between the years 1980 and 2006, it was estimated that 251 athletes among 690 diagnosed with a primary cardiovascular disease died of sudden cardiac death due to HCM (Maron *et al.* 2009). In 2015, the first study to screen for sarcomeric gene mutations was conducted on a cohort of 102 unrelated young Japanese athletes with abnormal ECG findings (Kadota *et al.* 2015). Heterozygous Arg106Trp and Thr1046Met mutations were identified in *MYBPC3* in four of 102 athletes, with two patients for each of the mutations. Genetic analysis on a 17-year-old football player with an abnormal ECG revealed an Arg495Trp mutation in *MYBPC3* (Martin *et al.* 2009). Other than the two studies mentioned here, athletes are generally not screened for mutations in sarcomeric genes and therefore the prevalence of specific *MYBPC3* mutations among young athletes is unknown. Since HCM is generally an inherited autosomal dominant disease, it is likely that athletes at risk for sudden cardiac death due to HCM carry a familial mutation, raising the question issue of why the parents also heterozygous for the mutation are not affected.

Not only is HCM the most common genetic heart disorder in people, it is the most common cardiac disease in cats, with an estimated prevalence of 10–15% (Payne *et al.* 2015; Freeman *et al.* 2017). HCM in cats is also a predominantly autosomal dominant disorder. Variation in clinical features among cats with HCM is extensive, from no disease symptoms to heart failure and sudden death. HCM is a common cause of congestive heart failure in cats and is prevalent among five breeds (Trehiou-Sechi *et al.* 2012), including the Maine Coon (Kittleson *et al.* 1999; Meurs *et al.* 2005; MacDonald *et al.* 2007) and Ragdoll (Meurs *et al.* 2007; Borgeat *et al.* 2014) breeds. Two mutations resulting in amino acid substitutions in cMyBP-C—Ala31Pro (A31P) (Meurs *et al.* 2005) and Arg820Trp (R820W) (Meurs *et al.* 2007)—have been associated with feline HCM in Maine Coon and Ragdoll cats, respectively. The feline and human cMyBP-C share 91.3% protein identity, as calculated using CLUSTAL multiple sequence alignment by MUSCLE (version 3.8; https://www.ebi.ac.uk/Tools/msa/muscle/). The A31P amino acid substitution in Maine Coon cats (Meurs *et al.* 2005) is near the N-terminus C0 domain of the cMyBP-C protein. This domain is predicted to interact directly with actin to promote activation of contraction by shifting tropomyosin on the thin filament. It has been postulated that the A31P substitution disrupts the interaction of the C0 domain with actin or the thin filament thereby affecting systolic or diastolic function (van Dijk *et al.* 2016). The R820W amino acid substitution in Ragdoll cats (Meurs *et al.* 2007) is located in the C6 domain of cMyBP-C, but the function of this domain is currently unknown. Secondary structure analysis predicts that the R820W mutation would result in increased hydrophobicity and disruption of the α-helical structure in this region of the protein (Meurs *et al.* 2007).

In both humans and cats, mutations in *MYBPC3* associated with HCM have highly variable effects, which could be caused by genetic background modifier loci and/or genotype by environment interactions (*e.g.* diet or exercise). Genes that are differentially transcriptionally co-regulated between wild-type (WT) and mutant *MYBPC3* alleles are candidate modifier loci (Anholt *et al.* 2003). In addition, genes that are differentially transcriptionally co-regulated between WT and mutant *MYBPC3* alleles give insight into the widespread molecular consequences of the mutation, which can be useful in developing therapeutic strategies (Vu Manh *et al.* 2005). Here, we developed a Drosophila model of feline HCM to assess the effects of the A31P and R820W mutations on organismal phenotypes and genome-wide gene expression by expressing WT and mutant feline *MYBPC3* alleles in the fly cardiovascular system using the *Gal4/UAS* binary expression system (Fischer *et al.* 1988; Brand and Perrimon 1993). There is currently no experimental evidence for Drosophila orthologs of mammalian *MYBPC3*. We therefore cloned the feline *MYBPC3* gene for integration into the Drosophila genome by *PhiC31* transformation (Groth *et al.* 2004). Our control for possible effects of global mis-regulation of an exogenously expressed gene is to compare the effects of the missense variants with the WT and background control strains. Vu Manh *et al.* (2005) previously used the *Gal4/UAS* system to express the human cMyBP-C protein (WT and two C-terminal truncated alleles) in Drosophila indirect flight muscle (IFM). Since striated IFM muscle fibers are highly ordered, any structural disturbances can be readily detected. The authors showed that the protein accumulated into the IFM sarcomeres, interacted with the endogenous Drosophila sarcomeric proteins leading to structural and morphological alterations of the myofibrils and to fiightless flies. Their study provides evidence that expression of mammalian *MYBPC3* produces a viable Drosophila model for cardiovascular disease.

Drosophila models have been successfully developed for numerous human diseases, including diabetes (Alfa and Kim 2016), glaucoma (Carbone *et al.* 2009), retinal degeneration (Colley 2012), Alzheimer’s disease (Fernandez-Funez *et al.* 2015), sleep disorders (Donelson and Sanyal 2015), and obesity (Smith *et al.* 2014). Fly models of cardiovascular disease emerged over 20 years ago with the development of assays to measure heart function and development (Bodmer 1993; Ocorr *et al.* 2014). Many cardiac related genes are evolutionarily conserved between Drosophila and humans including those involved in heart development (Bodmer and Venkatesh 1998; Vogler and Bodmer 2015), cardiac function (Medioni *et al.* 2009; Kooij *et al.* 2014), ion channels (Ocorr *et al.* 2007; Cavaliere and Hodge 2011), and contractile function (Cammarato *et al.* 2011; Chakraborty *et al.* 2015). It is likely that these conserved genes may be functioning in common physiological pathways, enabling an ideal translational model for vertebrate cardiac disease.

The insect heart consists of a hollow muscular tube that runs from the posterior abdomen into the thorax. The hemolymph (*i.e.* insect “blood”) flows freely within the body cavity where it makes direct contact with internal tissues and organs. Both the mammalian and insect hearts are divided into chambers that are separated by tiny valves through which fluid (blood or hemolymph) enters the heart. In insects, each of the four chambers contains six myocardial cells that facilitate the flow of hemolymph through the dorsal vessel. The aorta is the tube composed of myocardial cells that transports the hemolymph from the head into the body cavity (Ponzielli *et al.* 2002; Bier and Bodmer 2004; Zhu *et al.* 2016). Since other invertebrate genetic models such as *C. elegans* lack a heart, Drosophila presents the best simple model organism for translational studies on cardiac disease.

## Materials and methods

### Drosophila culture

All flies were maintained on cornmeal-agar-molasses medium at 25°C and 70% humidity, and a 12:12 h light:dark cycle.

### Transgenic lines expressing feline *MYBPC3*

The HCM *MYBPC3* variants were cloned from the cDNA of feline heart tissue (generously donated by Dr. Kathryn Meurs; NC-State University) to the *pUAST-attb* donor expression vector [which contains the *white*+ (*w*+) marker] using standard techniques, and subsequently validated by Sanger sequencing (Sanger *et al.* 1977; Bischof *et al.* 2007). The WT *MYBPC3* clone was used as the parental template for site-directed mutagenesis to obtain the pathogenic variants (A31P and R820W) using appropriate primer pairs (Supplementary Table S1). The variant clones were validated by Sanger sequencing to ensure that the desired mutagenesis had occurred (Supplementary Table S1 and Supplementary Figure S1). All *MYBPC3*-*pUAST-attb* constructs were submitted for *PhiC31* transformation (Groth *et al.* 2004) to Model System Injections (Durham, NC, USA). Transgenic flies bearing the WT, A31P, and R820W *MYBPC3*-*pUAST-attb* constructs were generated at a common integration site using *PhiC31* transformation (Groth *et al.* 2004) in the *y, w/P[int, y+]; Chr2/Chr2; P[*attP2*, y+]* isogenic genotype. Generation-0 (G0) flies were crossed to a white-eyed double-balancer stock (*w^1118^; CyO/Sp; Tm3, Sb/H*) and screened for transgenic F1 flies with red eyes. Homozygous transgenic lines were established with the isogenic genotype *w^1118^; Chr2; UAS-w+-MYBPC3*. We next expressed feline *MYBPC3* in the cardiovascular system of the fly using the *Gal4/UAS* binary expression system (Fischer *et al.* 1988; Brand and Perrimon 1993) by crossing the homozygous transgenic lines to a line homozygous for a cardiac-specific *Gal4* driver (*TinCΔ4-Gal4*) (genotype: *y, w; +/+; TinCΔ4-Gal4/TinCΔ4-Gal4*) (Lo and Frasch 2001). The three genotypes expressed in cardiac tissue are *w^1118^/y, w; Chr2/+; UAS-WT-MYBPC3/TinCΔ4-Gal4* (WT), *w^1118^/y, w; Chr2/+; UAS-A31P-MYBPC3/TinCΔ4-Gal4* (A31P), and *w^1118^/y, w; Chr2/+; UAS-R820W-MYBPC3/TinCΔ4-Gal4* (R820W). The control genotype is *w1118/y, w; Chr2/+; attP2/TinCΔ4-Gal4* (attP2). All transgenic alleles and the *attP2* genotype are co-isogenic, and when crossed to the same *Gal4* driver genotype, only differ in whether the feline *MYBPC3* alleles are expressed in cardiac tissue. There is no issue with variable genetic backgrounds that would require additional control crosses.

We used RT-PCR and RNA-sequencing to validate the expression of feline *MYBPC3* in larvae and young adult flies. For the RT-PCR, total RNA was extracted from 10 flies per sex per genotype using Qiazol (Qiagen), and the total RNA was converted to cDNA using the iScript cDNA synthesis kit (Bio-Rad). The cDNA from each sample was used as a template for the PCR reactions to amplify a 164-bp region of feline *MYBPC3*. As a control for the RNA integrity, we also amplified a 173-bp region of Drosophila 18 s RNA for each sample. Primer sequences are provided in Supplementary Table S1. PCR amplification was performed using Apex RedTaq (Genesee Scientific) with the following cycling parameters: 1 cycle of 95°C/2 min followed by 30 cycles of [95°C/30 s + 55°C/30 s + 72°C/30 s] and a final extension step of 72°C/4 min. The products were visualized on a 2% agarose gel. To further validate expression of feline *MYBPC3* in our fly model, the unaligned reads from our RNA-sequencing data (see “Genome wide expression profiling” section below) were mapped to the *MYBPC3* transcript (NCBI Accession: NM_000256.3). The read counts for *MYBPC3* of the WT-*MYBPC3*, A31P-*MYBPC3*, and R820W-*MYBPC3* samples were compared to the *attP2* control sample.

### Heart rate assays

The genetic architecture of numerous complex traits in Drosophila is typically sexually dimorphic (Williams and Carroll 2009). Sexual dimorphism and genetic variation in sexual dimorphism have been documented for morphological (Kopp *et al.* 2000), behavioral (Swarup *et al.* 2013; Carbone *et al.* 2016), physiological (Jordan *et al.* 2006; Harbison *et al.* 2013), and life history traits (Nuzhdin *et al.* 1997; Ivanov *et al.* 2015) in *D. melanogaster*. Therefore, we assessed the effect of the *MYBPC3* transgenes and the control separately for adult males and females and for larvae, as appropriate for each assay. In addition, all assays were conducted at the same time of day to avoid confounding effects of circadian rhythms.

Larval heart rate can be readily measured in transparent third instar larvae, allowing for the direct visualization of the heartbeat in intact animals. The fly heartbeat consists of a cardiac cycle that includes diastolic and systolic periods (Wasserthal 2007). We performed heart rate assays on 30 third-instar larvae from each of the three genotypes expressing *MYBPC3* in cardiac tissue (*w^1118^/y, w; Chr2/+; UAS-WT-MYBPC3/TinCΔ4-Gal4, w^1118^/y, w; Chr2/+; UAS-A31P-MYBPC3/TinCΔ4-Gal4, w^1118^/y, w; Chr2/+; UAS-R820W-MYBPC3/TinCΔ4-Gal4*) and the control genotype (*w1118/y, w; Chr2/+; attP2/TinCΔ4-Gal4*). Larval heart rate was measured by mounting third instar larvae onto a glass slide with double-sided tape so that their dorsal side faced upwards and their trachea were visible. Mounted larvae were then placed under a light microscope under 20X magnification and illuminated from below, and the heart rate was measured by counting the number of beats (*i.e.* pulses of the trachea) within a 15 s interval (Cooper *et al.* 2009). The movement of the trachea occurs due to pulling of the attachments from the heart (dorsal vessel). The heart rate measurement was repeated for four 15 s intervals per individual. Each heart rate within a 15 s interval is multiplied by 4 to calculate the beats per minute (BPM). Tukey and *T*-tests were performed using JMP^®^ Pro, Version 14 (SAS Institute Inc., Cary, NC, USA, 1989–2019).

### Climbing and exercise assays

On the day before the assay, flies of the three genotypes expressing *MYBPC3* in cardiac tissue and the control genotype were sorted by sex (50 flies/sex/line/replicate) and allowed to recover overnight from exposure to CO_2_. Three biological replicates/sex/line were assayed. To measure their inherent climbing ability, we used the countercurrent distribution apparatus designed by Benzer (1967). Flies were placed in their start-tube, gently tapped to the bottom of the tube and allowed 15 s to climb vertically. The apparatus was shifted once to the right and gently tapped such that any flies that climbed were collected into the neighboring tube. The flies were again allowed to climb vertically for 15 s after shifting the apparatus back to its start position. The process was repeated for a total of 7 climbing opportunities. At the end of the assay, the eight tubes containing flies were removed and each fly assigned a score from 1 (did not climb) to 8 (climbed seven times). The mean climbing score for each replicate was calculated as: ∑(*i × Ni*)/∑*Ni*, where *Ni* is the number of flies in the *ith* tube.

The flies were then returned to their original vial containing food and allowed to recover overnight. The following day, the flies were exposed to an intense exercise regime. The flies were transferred to empty vials and placed vertically in a rack secured to a shaker (VWR Signature™ High-Speed Microplate Shaker). The shaker was programmed to pulse for 10 s (600 rpm), causing the flies to fall to the bottom of the fly vial, followed by a 10 s rest period. During the 10 s rest period, the flies “exercise” by climbing upwards as they are innately negatively geotactic. The process was repeated for 1 h. At the end of the exercise regime, the flies were again scored for their climbing ability using the countercurrent distribution apparatus. Flies were then frozen at −80°C for RNA-sequencing. Standard least squares and Tukey test analyses were performed using JMP^®^ Pro, Version 13 (SAS Institute Inc., Cary, NC, USA, 1989–2019) using the full model: *Y* = *µ* + *G* + *S* + *T *+ G*S + G*T + S*T + G*S*T + *Rep*(G*S*T) + *ε*, and the reduced model (by sex): *Y* = *µ* + *G *+ *T *+ G*T  +  *ε*, where *G*, *S*, *T*, and *Rep* are the genotype (control, WT, A31P, and R820W), sex (M or F), treatment (exercised *vs* nonexercised), and replicate, respectively.

### Genome wide expression profiling

Larvae and 1-week old adult flies from the three genotypes expressing *MYBPC3* in cardiac tissue and the control genotype were collected over CO_2_ and allowed to recover from exposure to CO_2_ for 24 h prior to being flash-frozen for RNA-sequencing. In addition, 1-week old adult flies were also collected and flash frozen for RNA-sequencing after exposure to the exercise regimen described above. The samples were stored at −80°C until ready to process. Samples were processed for two biological replicates each of 10 pooled males and 10 pooled females from each genotype under normal rearing conditions and before and after 1 h of exercise, and for two biological replicates of 10 pooled larvae from each genotype, for a total of 40 samples.

Total RNA was extracted with QIAzol lysis reagent (Qiagen) and the Quick-RNA MiniPrep Zymo Research Kit (Zymo Research). Ribosomal RNA (rRNA) was depleted from 5 μg of total RNA using the Ribo-ZeroTM Gold Kit (Illumina, Inc). Depleted mRNA was fragmented and converted to first-strand cDNA using Superscript III reverse transcriptase (Invitrogen). Second strand cDNA was synthesized using dUTP instead of dTTP to label the second strand cDNA. cDNA from each sample was used to produce barcoded cDNA libraries using NEXTflexTM DNA barcodes (Bioo Scientific) with an Illumina TruSeq compatible protocol. Briefly, each sample was subjected to end-repair (Enzymatics), adenylation of 3’-ends (Enzymatics), and ligation of indexed adapters (Enzymatics and Bioo Scientific). Each enzymatic reaction was purified using 1.8X Agencourt AMPure XP beads (Beckman-Coulter). Size selection of each library was performed using Agencourt AMPure XP beads (Beckman Coulter) to an approximate insert size of 130 bp and a total library size of ∼250 bp. Second strand cDNA was digested with Uracil-DNA Glycosylase prior to PCR-enrichment to produce directional cDNA libraries. PCR-enrichment of the purified barcoded DNA was carried out with KAPA HiFi Hot Start Mix (Kapa Biosystems) and NEXTflex Primer Mix (Bioo Scientific). Libraries were quantified using the Qubit dsDNA HS kit (Life Technologies) and their sizes determined with the 2100 Bioanalyzer (Agilent Technologies). Each sample was diluted to equal molarity, quantified, multiplexed, denatured, and diluted to 15 pM. Clonal clusters for each pooled library sample were generated on the Illumina cBot and then sequenced on the Illumina HiSeq2500 using 125 bp single-read v4 chemistry (Illumina Inc.). We generated multiplexed libraries containing 12 samples each. Each multiplexed library loaded on one lane of the HiSeq2500.

We demultiplexed the RNA sequences using the Illumina bcl2fastq program; assessed read quality using the FastQC program and pre-processed reads using Cutadapt (Martin 2011) to remove residual adapter sequences. Residual ribosomal RNA (rRNA) sequences were removed following alignment of sequencing reads to known rRNA sequences using TopHat (Trapnell *et al.* 2009). All remaining reads were processed using the CLC Genomics Workbench 11 (https://www.qiagenbioinformatics.com/) which is based on previously published methods (Mortazavi *et al.* 2008). Reads were aligned to the *D. melanogaster* reference genome release 6.13 (Flybase.org). Unaligned reads were mapped to the *MYBPC3* transcript (NCBI Accession: NM_000256.3). Read counts and differential expression analyses were assessed using the CLC Genomics Workbench 11. Principal component analysis (PCA) was conducted in JMP^®^ Pro, Version 14 (SAS Institute Inc., Cary, NC, USA, 1989–2019). Gene expression changes were investigated for male and female transgenic flies (*attP2* control, WT, A31P, and R820W) under nonexercised and exercised conditions. PCA was performed using normalized read counts of the total number of expressed genes (∼16,000 genes).

Differential expression analyses were conducted for multiple comparisons to uncover differences due to expression of mutant *MYBPC3* under exercised and nonexercised conditions, for males, females, and larvae. We identified genes that were significantly differentially regulated at a false discovery rate (FDR) < 0.05 and with Log2FC > 0.5 or Log2FC < −0.5. Heat maps were generated using the online tool Heatmapper (http://www2.heatmapper.ca/expression/). To place the differentially expressed genes in their biological context, the following online bioinformatics tools for gene ontology, pathway analysis and identification of human orthologs were used: The Reactome Pathway Portal (www.reactome.org), Network Analyst STRING algorithm (www.networkanalyst.ca) (Szklarczyk *et al*. 2015), DAVID (https://david.ncifcrf.gov/), PANTHER Pathway (www.pantherdb.org/pathway/), and FlyBase (www.flybase.org). These tools allowed us to place feline candidate genes in a functional context by assigning biological processes to candidate genes associated with HCM risk.

### Knockdown of candidate genes using RNAi

We used RNA interference (RNAi) to knockdown the expression of candidate genes implicated by our differential expression analyses. Homozygous *UAS-RNAi* lines (*Act57B, Act79B, Act88F, Ef1alpha100E, Hsp22, Mhc, Mlc2*, and *TyrR*) were obtained from the Vienna Drosophila Resource Center (VDRC) (Dietzl *et al.* 2007). These flies were crossed to flies containing either the ubiquitin driver (*Ubi156-Gal4*) or the cardiac-specific *Gal4* driver (*TinCΔ4-Gal4*) to disrupt their gene expression. The progenitor genotypes (VDRC lines 60100 and 60000) were also crossed to both drivers as a control for lines from the KK[*PhiC31*] or GD[*P-element*] RNAi libraries, respectively. Larvae heart rates of the F1 progeny were measured as described above.

### Data availability

Supplementary data have been uploaded to the GSA Figshare portal. The expression data are deposited in the Gene Expression Omnibus under the accession ID GSE141574. Supplementary Figure S1 shows the Sanger sequencing chromatograms of the A31P and R820W mutations generated by site-directed mutagenesis. Supplementary Figure S2 shows the RNA expression of feline *MYBPC3* in our Drosophila model for feline HCM, using both RT-PCR and RNA-seq data. Supplementary Figure S3 shows the volcano plots of the differential expression analysis of genes in the *MYBPC3* transgenic lines (WT, A31P, and R820W) compared to the control line (*attP2*). Supplementary Figure S4 shows the principal component analyses of the RNA-seq data. Supplementary Figure S5 shows the protein–protein interaction (PPI) networks that were generated for significantly differentially expressed genes. Supplementary Figure S6 shows larval heart-rates resulting from RNA interference knockdown. Supplementary Table S1 lists the primer sequences used for cloning, site-directed mutagenesis, RT-PCR and Sanger sequencing. Supplementary Table S2 shows the results of the statistical analysis for the exercise assays. Supplementary File S1 is the gene expression browser for the RNA-seq data. Supplementary File S2 is the results of the differential expression analysis. Supplementary File S3 provides the data to generate the heat maps. Supplementary File S4 lists the gene ontology-biological processes (GO-BP) for the significantly differentially expressed genes.

Supplementary material is available at figshare DOI: https://doi.org/10.25387/g3.13228268.

## Results

### Expression of feline *MYBPC3* alleles in Drosophila

HCM is the most common heart disease in cats. Two *MYBPC3* gene mutations, A31P and R820W, were previously identified in the Maine Coon and Ragdoll breeds, respectively. To understand the disease etiology associated with these variants, we used the *Gal4/UAS* system to express feline *MYBPC3* in the fly cardiovascular system. We cloned the WT and variants (A31P and R820W) of feline *MYBPC3* to the *pUAST-attb* expression vector and generated Drosophila *UAS*-*MYBPC3* transgenic lines through *PhiC31* transformation. Positive transformants were crossed to a heart-specific driver line (*TinCΔ4-Gal4*) resulting in three co-isogenic genotypes expressing *MYBPC3* in cardiac tissue (*w^1118^/y, w; Chr2/+; UAS-WT-MYBPC3/TinCΔ4-Gal4, w^1118^/y, w; Chr2/+; UAS-A31P-MYBPC3/TinCΔ4-Gal4, w^1118^/y, w; Chr2/+; UAS-R820W-MYBPC3/TinCΔ4-Gal4*) and the control genotype (*w1118/y, w; Chr2/+; attP2/TinCΔ4-Gal4*). These genotypes are denoted as WT, A31P, R820W, and *attP2*, respectively, throughout the text. The lines were validated for gene expression of feline *MYBPC3* using RT-PCR and the normalized counts from the RNA-sequencing data for larvae, males, and females (Supplementary Figure S2). As expected, the three *MYBPC3* genotypes (WT, A31P, and R820W) show feline *MYBPC3* gene expression while the control line (*attP2*) does not, under both exercised and nonexercised conditions. We assayed these lines for their heart rates and exercise endurance and performed whole-transcriptome profiling using RNA-sequencing.

### Effects of feline variants of *MYBPC3* on heart rate and exercise endurance

**Figure 1:**
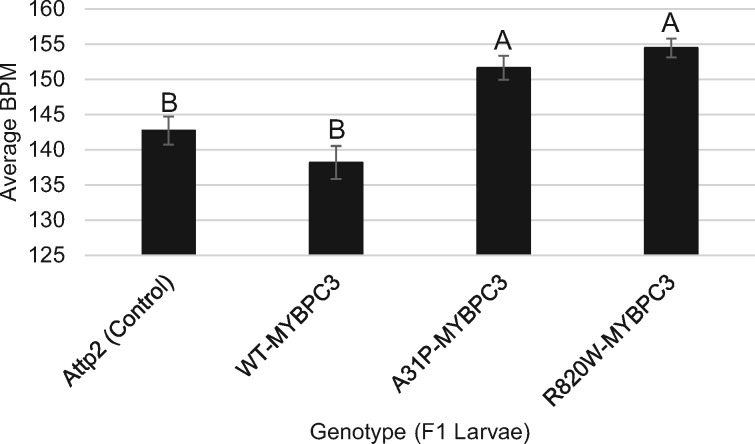
Heart rate of *D. melanogaster* larvae that express feline *MYBPC3*. Heart rates (BPM) of wild type (WT, *w^1118^/y, w; Chr2/+; UAS-WT-MYBPC3/TinCΔ4-Gal4*), A31P (*w^1118^/y, w; Chr2/+; UAS-A31P-MYBPC3/TinCΔ4-Gal4*), and R820W (*w^1118^/y, w; Chr2/+; UAS-R820W-MYBPC3/TinCΔ4-Gal4*) *MYBPC3* alleles expressed in cardiac tissue and the attP2 control (*w1118/y, w; Chr2/+; attP2/TinCΔ4-Gal4*). We tested all pairwise comparisons among means using the Tukey HSD test. Tukey groupings (A or B) are shown above each bar to indicate significantly different heart-rates. Tukey grouping A has a significantly different heart rate compared to Tukey grouping B.

We measured heart rates from larvae expressing feline WT and mutant *MYBPC3* alleles in the Drosophila cardiovascular system using a heart-specific *Gal4* driver. We observed a significant increase in heart rate of the A31P (*P *=* *2.7 × 10^−3^) and R820W (*P* = 2.5 × 10^−4^) mutant alleles compared to the WT *MYBPC3* WT allele (Figure 1). Importantly, there was no significant difference in heart rate between the control genotype and the *MYBPC3* WT allele, indicating that expressing the WT feline *MYBPC3* gene has no detectable effect on heart rate.

**Figure 2:**
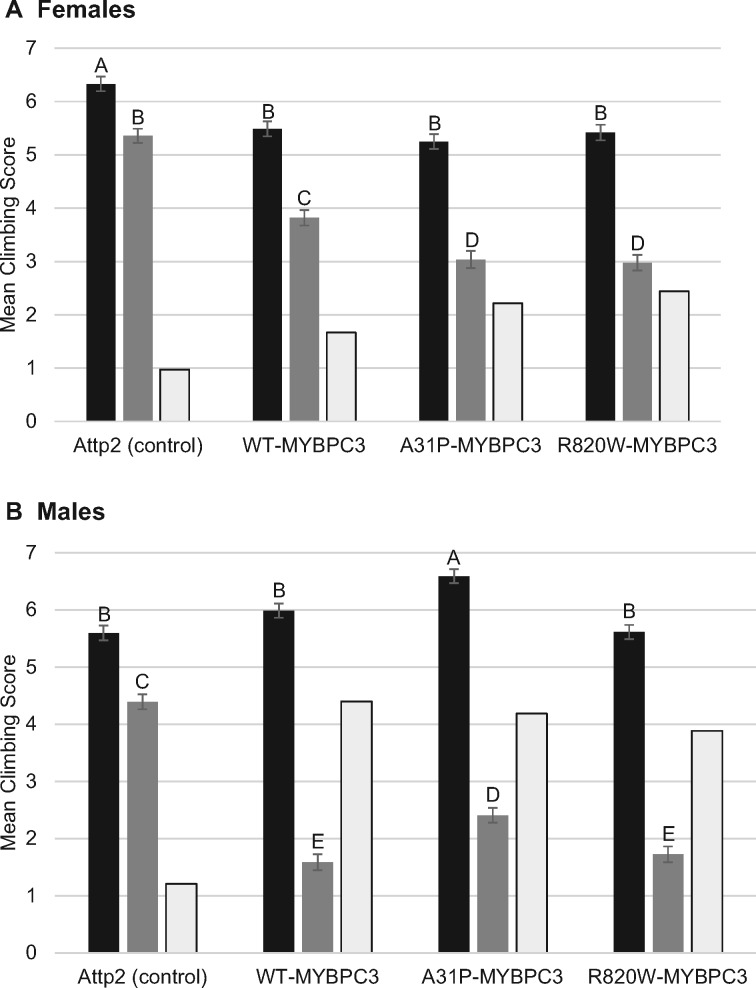
Climbing behavior before and after exercise. Climbing behavior before (black bars) and after (grey bars) 1 h of intense exercise of one week old *MYBPC3* alleles expressed in cardiac tissue [*w^1118^/y, w; Chr2/+; UAS-WT-MYBPC3/TinCΔ4-Gal4* (WT), *w^1118^/y, w; Chr2/+; UAS-A31P-MYBPC3/TinCΔ4-Gal4* (A31P), *w^1118^/y, w; Chr2/+; UAS-R820W-MYBPC3/TinCΔ4-Gal4* (R820W)] and their control [*w1118/y, w; Chr2/+; attP2/TinCΔ4-Gal4* (attP2)]. White bars indicate the difference between pre- and post-exercise. (A) Females. (B) Males. We tested all pairwise comparisons among means using the Tukey HSD test (by sex). Tukey HSD groupings (A–E) are indicated above each bar to indicate means that are significantly different from one another. Results of these statistical analyses are given in Supplementary Table S2.

We assessed the cardiovascular endurance of the transgenic lines by performing a baseline climbing assay and a second climbing assay immediately following 1 h of intense exercise (Figure 2, Supplementary Table S2). Climbing ability was significantly reduced following exercise for all genotypes in both sexes, with the greatest reductions among the genotypes expressing feline *MYBPC3*. The climbing ability of the pre-exercised flies was only moderately significantly different between the four genotypes in either sex. However, following the exercise regime, the A31P and the R820W genotypes showed a significant decrease in climbing ability compared to the *MYBPC3* WT genotype in both sexes, and all three *MYBPC3* genotypes had reduced climbing ability compared to the control post-exercise. This decrease was much greater in males than females.

### Gene expression changes due to expression of *MYBPC3* alleles

We quantified genome wide gene expression in flies that express the three *MYBPC3* transgene alleles (WT, A31P, and R820W) in the heart and for the control genotype (attP2) using RNA sequencing. Although we expressed feline *MYBPC3* specifically in the cardiovascular system of the fly, we measured genome-wide transcription of the whole organism. Therefore, any differential effects caused by expression of the *MYBPC3* alleles may ultimately cause downstream effects of other genes, across different tissues.

**Figure 3:**
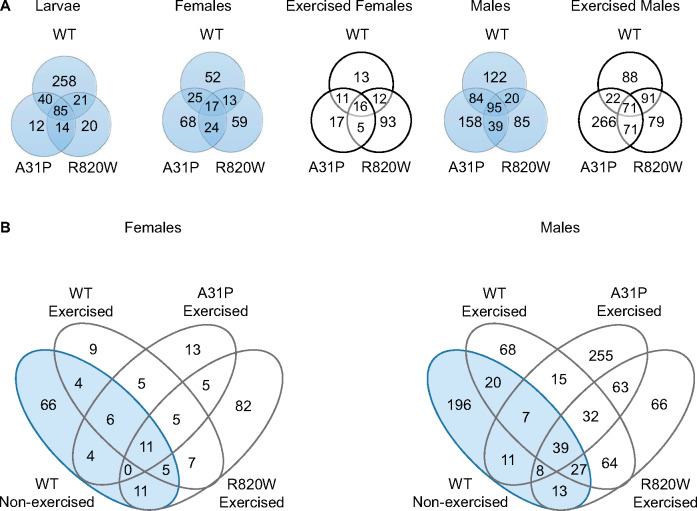
Numbers of overlapping or unique differentially expressed genes. The numbers of genes in the Venn diagrams represent significantly differentially expressed genes (FDR < 0.05 and Log2FC > 0.5 or Log2FC < −0.5) from pairwise comparisons of each feline *MYBPC3* allele expressed in cardiac tissue and the control genotype (A, top panel) or when the WT nonexercised genotype was compared to the exercised WT, A31P and R820W genotypes (B, bottom panel). Venn diagrams were generated using Venny 2.1 (https://bioinfogp.cnb.csic.es/tools/venny/). The nonexercised conditions are shown in blue and the exercised conditions are in white.

**Figure 4:**
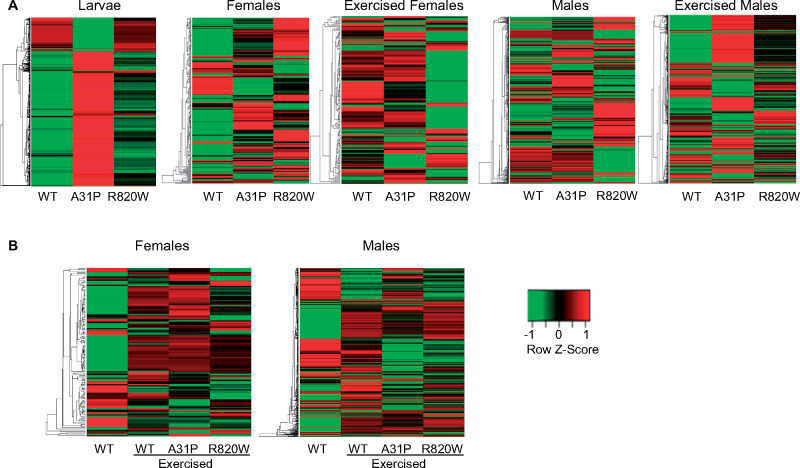
Heat maps of differentially expressed genes. Heat maps of differentially expressed genes among the *MYBPC3* alleles and the *attP2* control, expressed in cardiac tissue. The row *Z*-scores were calculated as *Z* = (*x*–*μ*)/*σ* where *x* is gene expression expressed as Log2FC; *μ* is the mean Log2FC across the samples; and *σ* is the standard deviation across samples for a given gene. Each row represents a significantly differentially expressed gene. Each column is the *MYBPC3* genotype normalized against the *attP2* control line. The heat maps were generated using www.heatmapper.ca. (A) Top panel shows heat maps for larvae and the nonexercised and exercised conditions for females and males. (B) Bottom panel shows heat maps comparing the nonexercised WT and exercised WT, A31P and R820W for both females and males.

We assessed gene expression in third instar larvae and in the 1-week-old males and females before and after exposure to an intense exercise regime (Supplementary File S1). We performed pairwise analyses of differential gene expression for each of the three *MYBPC3* transgenic lines compared to the control, separately for larvae and for exercised and nonexercised males and females (Supplementary File S2, Figure S3). We deemed genes to be significantly differentially expressed if the FDR < 0.05 and the Log2 fold change (Log2FC) was >0.5 (upregulated) or <−0.5 (downregulated). Table 1 lists the number of significantly differentially expressed genes among the transgenic lines by developmental stage, exercise treatment and sex. For each of the contrasts, many genes overlap between two or more *MYBPC3* alleles (Figure 3). A PCA was performed on the normalized RNA-seq read counts corresponding to the set of ∼16,000 expressed genes (Supplementary Figure S4). The first (60.2%) and second (13.2%) components represent most of the expression pattern. This analysis revealed a clear separation between males and females, by treatment (nonexercised *vs* exercised). Heat maps illustrating differential expression patterns of genes reveal clusters with differentially regulated patterns of expression between WT and the mutant lines (A31P and R820W) (Figure 4 and Supplementary File S3). To identify enrichment for genes with particular functions among those differentially expressed in response to *MYBPC3*, we tested the sets of significantly upregulated and downregulated genes identified from each transgenic line (by sex and condition) for GO-BP enrichment using Panther 14.1 (Supplementary File S4).

**Table 1:** Number of differentially expressed genes

	**SEX**	Larvae	Females	Females	Males	Males	
	**AGE**	Larvae	1-week	1-week	1-week	1-week	
	**Condition**	Not-exercised	Not-exercised	EXERCISED	Not-exercised	EXERCISED	
	**Total number of expressed genes**	15,965	15,290	15,370	16,224	16,352	
**Wild-type (WT)**	**Total**	365	107	52	321	272	**Number of significant genes**
	**Up-regulated**	62	32	29	142	147	
	**Down-regulated**	303	75	23	179	125	
**A31P**	**Total**	146	134	49	376	430	
	**Up-regulated**	20	73	31	181	283	
	**Down-regulated**	126	61	18	195	147	
**R820W**	**Total**	139	113	126	239	312	
	**Up-regulated**	25	79	36	91	156	
	**Down-regulated**	114	34	90	148	156	

Pairwise comparisons of differentially expressed genes of wild type (WT, *w^1118^*/*y*, *w*; *Chr2*/+; *UAS-WT-MYBPC3/TinCΔ4-Gal4*), A31P (*w^1118^*/*y*, *w*; *Chr2*/+; *UAS-A31P-MYBPC3/TinCΔ4-Gal4*) and R820W (*w^1118^*/*y*, *w*; *Chr2*/+; *UAS-R820W-MYBPC3/TinCΔ4-Gal4*) *MYBPC3* alleles expressed in cardiac tissue compared to the *attP2* control (*w^1118^*/*y*, *w*; *Chr2*/+; *attP2*/*TinCΔ4-Gal4).* Differential expression analysis was performed using CLC Genomics Workbench 11 (Qiagen). Significantly differentially expressed genes were selected based on FDR *P*-value < 0.05 and Log2FC > 0.5 (up-regulated) or Log2FC < −0.5 (down-regulated).

### Variants of feline *MYBPC3* elicit transcriptional responses to unfolded proteins in larvae

We identified 15,965 expressed genes among the WT, A31P, and R820W *MYBPC3* transgenic lines relative to the *attP2* control (Table 1) in larvae, with 365 (WT), 146 (A31P), and 139 (R820W) significantly differentially expressed genes. Among the significant genes, 88 were common to all three transgenic lines (Figures 3 and 4). The A31P and R820W larvae show significant enrichment of the following GO-BP categories compared to WT *MYBPC3*: response to heat (GO:0009408 and GO:0034605), response to unfolded proteins (GO:0006986 and GO:0034620), peptide metabolic process (GO:0006518), and chaperone-mediated protein folding (GO:0061077) (Supplementary File S4). These categories are represented by genes that include *Hsp70Ab, Hsp70Bb, Gba1a, CG8773*, and *CG8774*. Human HSC70 is the ortholog of Drosophila *Hsp70Ab/Hsp70Bb* and has recently been shown to serve as a chaperone for both WT and several mutant forms of *MYBPC3* protein using co-immunoprecipitation/mass spectrometry (Glazier *et al.* 2018). *Glucosylceramidase beta* (GBA) is the human ortholog of Drosophila *Gba1a*. Mutations in *GBA* have been associated with Gaucher disease (GD) type III in patients presenting with cardiovascular calcifications (Beutler *et al.* 1995; Chabas *et al.* 1995; George *et al.* 2001). Drosophila models for GD have been established to study the effects of mutant *Gba1* (Suzuki *et al.* 2015; Davis *et al.* 2016; Kinghorn *et al.* 2016; Cabasso *et al.* 2019). A Drosophila model for GD using two existing *GBA1* mutant fly lines recapitulated hallmarks of GD including shortened lifespan, neuroinflammation, and activation of the unfolded protein response (Cabasso *et al.* 2019).

The human ortholog of *CG8773/CG8774* is predicted to be *ENPEP*, a member of the renin-angiotensin system that controls blood pressure and hypertension; known risk factors for atrial fibrillation (AF) (Healey and Connolly 2003). *Enpep* is expressed in regions of the developing mouse heart essential for cardiac electrical activity (sinoatrial node and arrhythmogenic sites). Dys-regulation of *ENPEP* may contribute to an increased risk of AF in carriers of *PITX2* disease-associated variants (Aguirre *et al.* 2015).

Several clusters of genes are differentially regulated between the WT and mutant (A31P and R820W) transgenic lines (Figure 4). In general, the WT show clusters of downregulated genes while those same clusters are upregulated in the A31P and R820W lines. These clusters include genes involved in proteolysis (*Bace* and *Phae2*), response to unfolded protein (*Gba1a*), and heart development (*prc*). Pericardin (*prc*) is a Collagen IV-type protein essential for cardiac extracellular matrix formation and heart function in Drosophila (Chartier *et al.* 2002; Wilmes *et al.* 2018; Cevik *et al.* 2019) and warrants future exploration for its effect on heart morphology due to the presence of *MYBPC3* variants.

### Expression of feline *MYBPC3* in female Drosophila reveal distinct patterns of gene regulation between exercised and nonexercised flies, including a cluster of differentially regulated snoRNAs

We identified over 15,000 expressed genes when we performed RNA-sequencing on adult females of the transgenic and control genotypes and compared nonexercised flies to those that were exposed to a 1 h exercise regime: 15,290 expressed genes for nonexercised flies and 15,370 genes for exercised flies (Table 1). There were 107 (WT), 134 (A31P), and 113 (R820W) significantly differentially expressed genes for the nonexercised females, of which 17 were common to all three transgenic lines; and 52 (WT), 49 (A31P), and 126 (R820W) significantly differentially expressed genes for the exercised females, of which 16 were common to all three transgenic lines (Table 1, Figures 3 and 4). No gene ontology categories were significantly enriched for the WT or the R820W nonexercised females (Supplementary File S4). For the A31P nonexercised females, the following GO-BP categories were enriched: response to heat (GO:0009408 and GO:0034605), response to unfolded protein (GO:0006986, GO:0034620), chaperone-mediated protein folding (GO:0061077), and immune system process (GO:0002376). The protein-folding processes are represented mostly by genes encoding heat-shock proteins (*Hsp22, Hsp70Bb, Hsp70Bbb*, and *Hsp70Bc*). Heat-shock proteins are a family of evolutionary conserved proteins that function as molecular chaperones. These proteins recognize and form complexes with incorrectly folded or denatured proteins and assist with their correct folding or degradation (Jagla *et al.* 2018). It is conceivable that the *MYBPC3* A31P variant is misfolded, resulting in the up-regulation of the Hsp proteins possibly eliciting an immune response (Bolhassani and Agi 2019). When females from the transgenic lines are exercised, the same GO-BP categories as the nonexercised females are enriched, for the WT and A31P lines (response to heat, response to unfolded protein and chaperone-mediated protein folding); however, the only *Hsp* gene represented by these categories is *Hsp70Bb*. No GO-BP categories were enriched for the exercised R820W variant genotype.

**Figure 5:**
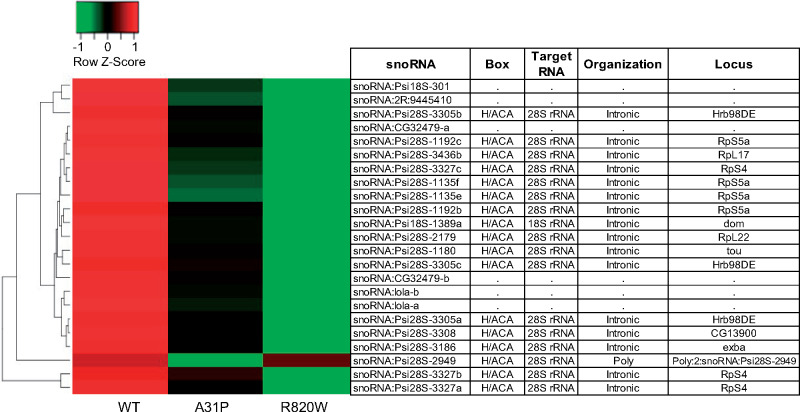
Heat maps of differentially expressed small nucleolar RNAs (snoRNAs) between feline *MYBPC3* alleles in exercised females. The row *Z*-scores were calculated as *Z* = (*x*–*μ*)/*σ* where *x* is gene expression expressed as Log2FC; *μ* is the mean Log2FC across the samples; and *σ* is the standard deviation across samples for a given gene. Each row represents a significantly differentially expressed snoRNA gene and each column is the *MYBPC3* genotype normalized against the *attP2* control line. The box type, target RNA, organization, and locus were identified using snOPY (http://snoopy.med.miyazaki-u.ac.jp/) (Yoshihama *et al.* 2013). No other condition exhibited co-regulated differential expression of snoRNAs. The heat maps were generated using www.heatmapper.ca.

Several clusters of differentially expressed genes were coordinately up- or down-regulated between the *MYBPC3* genotypes under each exercise regime. For nonexercised females, clusters of downregulated genes in WT were upregulated in A31P and R820W. These clusters include GO-BP categories such as carbohydrate metabolic process (*Mal-A1* and *Mal-A6*), transmembrane transport (*CG17751, CG6484, CG16727*, and *Mdr50*), oxidation-reduction process (*Cyp6a21*) and proteolysis (*Jon25Biii* and *ome*). In exercised females, clusters of genes were downregulated for R820W, but upregulated for WT and A31P. These clusters include GO-BP categories such as muscle system process (*Fhos*), myofibril assembly (*Act88F*) and a cluster of 23 small nucleolar RNAs (including *snoRNA-lola-a, snoRNA-lola-b*, and *snoRNA-Psi-28S*) (Figures 4 and 5). The snoRNAs are a class of small noncoding RNAs that guide post-transcriptional modifications such as the conversion of uridines into pseudouridines. Other functions of *snoRNAs* include regulation of mRNA editing, alternative splicing, and gene silencing (Bachellerie *et al.* 2002; Bratkovic and Rogelj 2014; Deogharia and Majumder 2018). A genome wide association study (GWAS) involving 5244 participants of the PROspective Study of Pravastatin in the Elderly at Risk (PROSPER) identified SNPs in the snoRNA cluster on chromosome 14q32 that were significantly associated with heart failure, and showed that snoRNA gene expression in this cluster is upregulated during cardiovascular disease (Hakansson *et al.* 2019). Altered expression of H/ACA snoRNAs has also been implicated in other human diseases including leukemia, prostate cancer and nonsmall cell lung cancer (for a review see, McMahon *et al.* 2015). It is conceivable that the snoRNAs identified by our study regulate the disease etiology of HCM.

### Exercise induces an enrichment of heat shock protein genes in male *MYBPC3* transgenic lines

**Figure 6:**
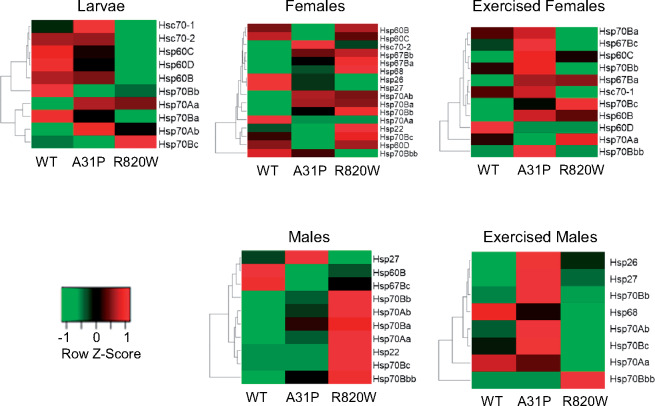
Heat maps of differentially expressed Heat shock proteins (Hsps) between feline *MYBPC3* alleles. The row *Z*-scores were calculated as *Z* = (*x*–*μ*)/*σ* where *x* is gene expression expressed as Log2FC; *μ* is the mean Log2FC across the samples; and *σ* is the standard deviation across samples for a given gene. Each row represents a significantly differentially expressed *Hsp* or *Hsc* gene. Each column is the *MYBPC3* genotype normalized against the *attP2* control line. The heat maps were generated using www.heatmapper.ca.

We identified over 16,000 expressed genes in adult males for each exercise regime (Table 1). A total of 321 (WT), 376 (A31P), and 239 (R820W) genes were significantly differentially regulated for the nonexercised males and 272 (WT), 430 (A31P), and 312 (R820W) genes were significantly differentially regulated for the exercised males. Among the significantly differentially regulated genes, 95 (nonexercised males) and 71 (exercised males) were in common between the WT, A31P, and R820W transgenic lines (Table 1, Figures 3 and 4). The top GO-BP categories for all three transgenic lines in unexercised males include defense response to bacteria (GO:0019731, GO:0050829, and GO:0050830), immune response (GO:0006959 and GO:0006955), response to heat (GO:0034605, GO:0009408, and GO:0034605), and response to unfolded protein (GO:0006986, GO:0034620, and GO:0061077). A comparison of differentially expressed genes in exercised and nonexercised male flies revealed that genes involved in the response to unfolded protein or to protein refolding (*Hsp70Aa, Hsp70Ab, Hsp70Ba, Hsp70Bb, Hsp70Bc*, and *Hsp70Bbb*) were uniquely enriched in the exercised flies (Figure 6).

Several clusters of differentially expressed genes were coordinately regulated between the *MYBPC3* genotypes under each exercise regime. A cluster of upregulated genes was induced by R820W expression in nonexercised males that were downregulated in WT and A31P, including genes involved in muscle contraction (*mlc1* and *Tm1*) and protein refolding (*CG14207* and *l(2)ef1*) (Figure 4D and Supplementary File S4). In addition, another cluster of genes was downregulated in R820W and upregulated in WT and A31P, including genes involved in G-protein coupled receptor signaling (*Msr1, Rh4, Rh5, Rh6, AkhR*, and *mthl3*), axon guidance (*tok, daw*, and *Lsp2*), and oxidation-reduction process (*Hpd, Cyp4ac1*, and *CG4335*). In the exercised males, there was a large, downregulated cluster for WT that is upregulated in the A31P line, but lowly-expressed in R820W (Figure 4E and Supplementary File S3). GO-BP processes within this cluster include proteolysis (*CG31928, CG6508, CG31926, ndl*, and *stet*), posttranscriptional gene silencing (*CG9925* and *Marf1*), DNA replication (*RnrL, Mcm10, Orc2*, and *PCNA*), and chorion-containing eggshell formation (*Cp7Fa, Cp7Fb, Cp7Fc, Muc12Ea*, and *dec-1*).

### Transcriptional responses to proteolysis and oxidation-reduction processes of exercised flies compared to nonexercised WT flies

We compared the gene expression profiles of nonexercised flies that express the WT-*MYBPC3* allele to exercised WT, A31P, and R820W *MYBPC3* flies (Supplementary Files S2 and S3, Figures 3 and 4). The comparison of nonexercised WT to exercised WT flies revealed 26 genes in females and 179 genes in males that were exclusively significantly differentially regulated when the flies underwent the exercise regime (Figure 3B). A major portion of these genes have biological processes relating to oxidation-reduction (*Cyp4ac2, Cyp6a8, Cyg6a2*, and *Cyp6a17*), mannose metabolic process (*LManI-lManVI*), proteolysis (*NepI9, Nep7*, and *Jon99Fi, Jon65Ai, Jon99Fii, Ser8*), and transmembrane transport (*Tret1-1, MFS1*, and *MFS14*). The top up-regulated genes include *asRNA: CR45271, CG17672, CG43773*, and *lncRNA: CR44080* while the top–down-regulated genes include *Hsp70Bbb, Hsp70Aa, ImpL1*, and *lncRNA: CR44602* and *phu*. Heat map analysis (Figure 4B and Supplementary File S3) revealed 37 genes (162 genes) that were up-regulated in female (male) nonexercised WT flies but down-regulated in exercised WT flies. The Panther Overrepresentation Test (Fisher’s Exact Test) of these genes revealed that response to incorrect/unfolded protein was significantly over-represented (FDR < 0.003). Conversely, 73 genes (213 genes) were down-regulated in female (male) nonexercised WT flies, but up-regulated when the WT flies were exercised. The following biological processes were significantly over-represented (FDR < 0.05) in this case; immune/defense response (FDR < 0.0036) and response to incorrect/unfolded protein (FDR < 0.008). These categories may indicate that exercise activates genes that reduce inflammation (*i.e.* immune response) and muscle damage (*i.e.* proteolysis) to assist with recovery post-exercise.

A31P exercised females showed 6 exclusively significantly up-regulated and 12 significantly down-regulated genes compared to the WT nonexercised females. In exercised A31P males, 50 were exclusively up-regulated and 220 were exclusively down-regulated compared to the nonexercised WT males. Cytochrome P450 genes were significantly down-regulated in both females (*Cyp6a17* and *Cyp6a2*) and in males (*Cyp6a19* and *Cyp6a2*). In females, two additional cytochrome P450 genes, *Cyp4p2* and Cyp6a8, were up-regulated. Down-regulated genes in the exercised A31P males represented a variety of functions including: cell death (*Drep-2*, *CG31928*, and *CG6508*), defense response (*Hsp27*, *CG9925*, *CBP*, and *Pebp1*), protein processing (*Bace*, *pcl*, *stg*, *CG18190*, *Cdk1*, *lok*, *Cdk2*, and *stet*), proteolysis (*CG32834* and *CG31681*), and oxidation-reduction process (*Nox*, *CG12398*, *RnrS*, *RnrL*, *Cyp6a19*, *Cyp6a2*, *PH4alphaPV*, and *CG4009*).

In the exercised female flies expressing R820W-*MYBPC3*, 17 genes were exclusively up-regulated and 72 were down-regulated compared to the WT-*MYBPC3* nonexercised flies. The down-regulated genes are involved in a variety of processes including proteolysis (*CG4998*, *gammaTry*, and *CG6067*), metabolic processes (*ldgf2*, *Tps1*, *CG6295*, *Gpo-1*, *Hml*, *verm*, and *CG9463*), and cell adhesion (*Dscam4*). Of the upregulated genes, 10 have unknown functions. Three genes (*Cp7Fa*, *Cp36*, and *Cp18*) are involved in multicellular organism development; two genes (*CG4595* and *CG4009*) are involved in oxidation-reduction processes; the gene *c(3)G* is involved in synaptonemal complex assembly; and *CG5966* is involved in lipid catabolic processes.

The exercised male flies expressing R820W-*MYBPC3*, 66 genes were exclusively up-regulated and 64 were down-regulated compared to the nonexercised WT-*MYBPC3*. Up-regulated genes included those involved in metabolic processes (*tobi*, *Cht4*, *Ect3*, *Amyrel*, *Mal-A7*, and *Mal-B2*); locomotion/flight (*CG31148* and *TpnC4*); oxidation-reduction process (*mt: ND3*, *su(r)*, *Gapdh1*, and *CG6910*), and proteolysis (*Phae1*). Down-regulated genes included those involved in proteolysis (*CG31233*, *CG17633*, *CG7025*, *Jon99Fi*, *CG11911*, *CG5246*, *Jon99Fii*, and *Ser8*); response to endoplasmic reticulum stress (*bgm*) and transmembrane transport (*CG4562*, *CG42269*, *CG8654*, *CG30272*, and *CG4822*).

Heat map analysis (Figure 4B) in females (males) revealed 2 (1) major clusters of genes that were down-regulated in nonexercised WT flies, but up-regulated among the exercised flies (WT, A31P and R820W). Included in these clusters are genes involved in muscle system processes (*fln, Neurochondrin, Mlc1, Mlc2, Mhc, Tm1, kon*, and *Fhos*), defense response (*BomS1, BomS2, BomS5, Dso2*, and *msopa*), and muscle contraction (*Act57B, Act79B*, and *Act88F*). In males, several up-regulated genes in nonexercised and exercised WT flies were down-regulated only in exercised A31P and R820W flies. These include 32 protein-coding (CG) genes with unknown biological process, genes involved in microtubule-based movement (*Sdic2, CG7276, CG8407*, and *CG10859*), and oxidation-reduction processes (*Cyp6a16, Gpo3*, and *TotX*). Six genes involved in proteolysis (*CG11034, CG11912, CG18179, CG18180, Jon25Biii*, and *Jon74E*) were down-regulated in WT (nonexercised and exercised), but up-regulated in exercised A31P and R820W males.

### Knockdown of candidate genes leads to arrhythmias

We used RNAi knockdown to functionally test whether reduced expression of candidate genes implicated by the differential expression analyses affect larval heart rates. We selected 8 candidate genes for RNAi mediated suppression of gene expression (*Act57B, Act79B, Act88F, Ef1alpha100E, Hsp22, Mhc, Mlc2*, and *TyrR)* specifically in the cardiovascular system of the fly using the *TinCΔ4-Gal4* driver line. Knockdown of five genes (*Act57B, Act79B, Mhc, Mlc2*, and *TyrR*) significantly decreased larval heart rate compared to the control line, while knockdown of two genes (*Ef1alpha100E* and *Hsp22*) significantly increased the heart rates (Supplementary Figure S6). The Homophila database (http://homophila.sdsc.edu) reports that the *Act* genes (*Act57B, Act79B*, and *Act88F*) and *Mlc2* are close matches to the human *ACTC1* gene (NP_005150) and *MYL2* (NP_000423) genes, respectively. Mutations in both these genes have been associated with familial HCM (van Waning *et al.* 2019; Manivannan *et al.* 2020). Next-generation sequencing of an Italian family with HCM revealed an Ala21Val mutation in *ACTC1* resulted in myofibrillar disarray causing dis-anchorage of myofilaments (Frustaci *et al.* 2018). These patients presented with palpitations and ventricular tachycardia due to structural defects of the myofilaments resulting from the Ala21Val mutation. In a Lebanese family presenting with congenital heart defects and arrhythmias, a Met84Thr mutation in *ACTC1* was identified by Sanger sequencing (Augière *et al.* 2015). The structural consequences of this mutation were examined using 3 D structural analysis and was found to reside in a region of the Actin filament in extremely tight apposition to the myosin head. Disease-causing *ACTC1* mutations can disrupt both electrostatic and hydrophobic contacts, resulting in perturbation of the interaction between Actin, Tropomyosin and Myosin (Augière *et al.* 2015). An RNAi screen in Drosophila primary cell culture of heart muscle disease genes associated with congenital myopathies and cardiomyopathies lead to abnormal muscle phenotypes in primary culture (Bai *et al.* 2008). For example, knockdown of *Mhc* and *Mlc2* (both genes are involved in the regulation of Myosin function) resulted in the lack of striation in both Actin and Myosin filaments. Knockdown of Actin isoforms including *Act57B* in primary cell culture resulted in thinner or shorter myofibril structures, possibly resulting from an arrest in myofibril assembly from the lack of available Actin monomers (Bai *et al.* 2008). RNAi knockdown of *Mhc* in Drosophila cardiomyocytes with the heart-specific *Hand-Gal4* driver line resulted in softening of the cardiomyocytes, due to reduction in myofibrillar density (Kaushik *et al.* 2011). In addition, the authors identified severe impairment of the heart tube with significant loss of rhythmic contractions. Results from our RNAi knockdown experiment together with the previous studies mentioned here demonstrate that these genes, when knocked-down or mutated, may result in myofibril structural defects resulting in cardiovascular defects.

## Discussion

HCM affects 10–15% of the pet cat population (Payne *et al.* 2015; Freeman *et al.* 2017). Most affected cats remain free of clinical signs; however, a proportion experience serious complications, including congestive heart failure, arterial thromboembolism, and sudden cardiac death, similar to human HCM (Paige *et al.* 2009; Payne *et al.* 2015). Breeds including Maine Coon, Ragdoll, Persian, and Sphynx are predisposed to HCM, suggesting a genetic component to the disease (Kittleson *et al.* 2015). Two missense mutations in *MYBPC3* were identified in the Maine Coon (A31P) and Ragdoll breeds (R820W) (Meurs *et al.* 2005, 2007). The molecular mechanism(s) by which mutations in *MYBPC3* give rise to HCM remains poorly understood, particularly given the broad spectrum of clinical manifestations, from benign or asymptomatic to sudden death. Studies of the genetic factors and biological mechanisms that underlie this disease are as challenging in felines as they are in humans, as they maintain extensive variation due to differences in factors including genetics, disease, medication, and diet. Model organisms can control for these factors. A powerful strategy for studying the molecular effects of vertebrate disease variants is to create transgenic *D. melanogaster* bearing the disease gene/variants and express them in cardiac tissue using the versatile *Gal4/UAS* system (Brand and Perrimon 1993; Carbone *et al.* 2009).

Here, we developed a transgenic fly model for feline HCM by expressing feline WT *MYBPC3* and two *MYBPC3* variants associated with disease (A31P and R820W) in the Drosophila cardiovascular system, and assessed the cardiovascular health and transcriptional consequences specific to each *MYBPC3* variant. Transcriptionally co-regulated genes in the presence of *MYBPC3* mutations relative to the *MYBPC3* WT allele are potential modifiers affecting the variable effects of *MYBPC3* mutations in different genetic backgrounds, and transcriptionally co-regulated genes that differ between exercised and nonexercised flies with the *MYBPC3* mutations are potential candidate genes affecting genotype by environment interactions affecting manifestation of disease.

One potential caveat for this approach is that there is no experimental evidence for Drosophila orthologs of mammalian *MYBPC3*. The DRSC Integrative Ortholog Prediction Tool (DIOPT) (https://www.flyrnai.org/cgi-bin/DRSC_orthologs.pl) identifies four Drosophila proteins (Unc-89, Sls, Hbs, and Sns) as potential orthologs, however, the DIOPT score for orthology is low (1/15) for each of these. Our control for possible effects of global mis-regulation of an exogenously expressed gene is to compare the effects of the missense variants with the WT feline gene and fly control genotype.

We measured exercise endurance and larval heart rates as a proxy to assess the cardiovascular health of our *MYBPC3* transgenic lines. Previously, RNAi knockdown of *Atg* genes in Drosophila muscle and heart led to impaired locomotor function and displayed an accelerated age-dependent loss of cardiac function (Xu *et al.* 2019). The flight ability and cardiac structure and function were also affected in a Drosophila model for HCM by expressing a mutation in human β-cardiac myosin heavy chain, known to cause HCM (Kronert *et al.* 2018). Several studies have shown that exercise training prevents age-related heart dysfunction in Drosophila (Piazza *et al.* 2009; Sujkowski *et al.* 2015; Zheng *et al.* 2017; Sujkowski and Wessells 2018; Blice-Baum *et al.* 2019). These results indicate that measurements of locomotor function are valuable for examining heart health in fly models for HCM. We therefore assessed whether the *MYBPC3* variants were able to mimic symptoms presented by patients diagnosed with HCM by assessing the cardiovascular health of the transgenic lines by quantifying heart rate and exercise endurance for each genotype.

We observed a significantly increased heart rate of both the A31P and R820W mutants compared to the WT (Figure 1). However, there was no significant difference in heart rate between the WT *MYBPC3* allele and the control genotype, showing that expression of the WT feline *MYBPC3* gene does not impair heart rate. Exercise assays for cardiovascular endurance showed that the A31P and R820W mutants had significantly reduced endurance post-exercise in both sexes (Figure 2).

Gene expression analyses using RNA-sequencing revealed significantly regulated genes that responded to the expression of feline *MYBPC3* variants. These included a cluster of 23 snoRNAs that were downregulated by R820W but upregulated by WT only in the exercised females. Fifteen of these snoRNAs are encoded by introns and belong to the box H/ACA class that guide pseudouridylation (conversion of uridine to pseudouridine) of the target 28S RNA (Yoshihama *et al.* 2013). Down-regulation of box H/ACA snoRNAs (such as *SNORA15* and *SNORA24*) has been associated with human diseases including dyskeratosis congenita (Bellodi *et al.* 2013) and leukemia (Valleron *et al.* 2012). When de-regulated, the snoRNAs identified by this study may be involved in the pathogenesis of HCM particularly under conditions of cardiac stress. The study of snoRNAs and their effect on human disease pathogenesis is an emerging field that could lead to the development of therapeutics that would modulate the expression of H/ACA snoRNA.

Genes encoding Heat shock proteins (Hsp and Hsc) were also de-regulated in this study. The *Hsp70* gene family are molecular chaperones that promote the refolding of misfolded proteins or suppress protein aggregation (Kettern *et al.* 2011; Rosenzweig *et al.* 2019), thus protecting the cell from deleterious proteotoxic effects. Here, we observed down-regulation of five *Hsp* genes in the R820W larvae (*Hsc70, Hsc70-2, Hsp60C, Hsp60D*, and *Hsp60B*) with concomitant up-regulation in the WT larvae. In adult flies, the genes *Hsp70Bbb* and *Hsp70Aa* were significantly down-regulated after WT flies experienced the exercise regime. Exercised A31P females and males show an overall up-regulation of several *Hsp* genes that are down-regulated in exercised R820W and WT transgenes. In a previous study (Glazier *et al.* 2018), co-immunoprecipitation analyses in a cardiomyocyte *MYBPC3* model for HCM showed that HSC70 is a chaperone for WT and mutant *MYBPC3*. HSC70 likely plays a role in maintaining cardiomyocyte proteostasis by assisting with the folding and turnover of *MYBPC3* (Glazier *et al.* 2018); however, further studies on its precise role in HCM are needed. A Drosophila model for AF showed that overexpression of Drosophila *Hsp23* was protective against contractile dysfunction and cardiac remodeling (Zhang *et al.* 2011). HSPB1 may protect from cardiac remodeling thus reducing the progression of human AF (Brundel *et al.* 2006; Yang *et al.* 2007). Drosophila, cardiomyocyte, and canine models of AF show that HSP-inducing compounds such as geranylgeranylacetone and their derivatives can prevent proteostasis and cardiac remodeling, thus attenuating AF progression (Brundel *et al.* 2006; Zhang *et al.* 2011; Hoogstra-Berends *et al.* 2012). Pharmacological induction of HSPs could potentially be considered for future studies in the treatment or prevention of HCM.

The cytochrome P450 (CYP) superfamily of enzymes are involved in the metabolism of fatty acids, xenobiotics, and therapeutic drugs. Several studies have shown that expression of CYP enzymes and therefore the production of endogenous metabolites are altered in patients with cardiovascular disease (Elbekai and El-Kadi 2006). The production of these endogenous CYP metabolites [epoxyeicosatrienoic acids (EETs), hydroxyeicosatetraenoic acids (HETEs), prostacyclin, and aldosterone] are involved in the maintenance of cardiovascular health, including the regulation of heart contractility (Aspromonte *et al.* 2014; Rowland and Mangoni 2014) . In a transgenic mouse model that overexpressed CYP2J2 in cardiomyocytes, cardioprotective EETs were generated that protected against arrhythmia susceptibility in cardiac hypertrophy (Westphal *et al.* 2013). On the other hand, elevated 20-HETE levels due to overexpression of CYP4A2 can lead to hypertension in rats (Wang *et al*, 2006). The results from our RNA-sequencing experiment when comparing the nonexercised WT-*MYBPC3* flies to the exercised WT, A31P and R820W lines, revealed the dysregulation of CYP genes in both male and female flies, further implicating a role for these enzymes in maintaining cardiovascular health.

A previous study (Vu Manh *et al.* 2005) used the *Gal4/UAS* system in Drosophila as a model to study effects of *MYBPC3* mutations associated with human familial HCM. Transgenic ﬂies carrying human WT or two C-terminal truncated cMyBP-Cs were expressed in IFM to study transcriptional responses and flight phenotypes. Although the study only tested 3570 genes using spotted microarrays, and expressed *MYBPC3* in flight muscle rather than cardiac tissue, many genes overlapped between our two studies including *Bace/CG13095* (proteolysis), *CG11796* (oxidation-reduction process), *CG11911* (proteolysis), *CG15818, fln* (sarcomere organization), *Hsp27* (chaperone-mediated protein folding), *Mal-A6* (oxidation-reduction process), *Prm* (myofibril assembly), and *Tollo* (signal transduction). The processes associated with these overlapping genes are found among several of the genes that were dysregulated in our study, indicating that common biological processes are modified by different mutations in *MYBPC3* known to cause HCM.

PPI networks derived using the differentially expressed genes revealed several hub proteins (degree > 10) (Supplementary Figure S5). Hub genes encoding these proteins include actins, which are involved in muscle contraction (Act57B, Act79B, Act87E, and Act88F); and the Hsps involved in protein folding (Hsp70Aa, Hsp70Bbb, and Hsp70Bc). The results of our RNAi knockdown experiments revealed that the hub genes *Act57B* and *Act79B* displayed significantly reduced heart rates compared to the control line when knocked down, further implicating these genes in the disease etiology of HCM. Males harboring the mutant alleles showed more highly connected PPI networks than females and include a range of differentially expressed genes encoding hub proteins involved in a wide range of biological processes, including DNA replication, protein phosphorylation and histone acetylation/phosphorylation.

Several exercise studies in Drosophila have been published that assessed physical fitness using instruments such as the Power Tower (Piazza *et al.* 2009), TreadWheel (Mendez *et al.* 2016), Swing Boat (Berlandi *et al.* 2017), and the Rotating Exercise Quantification System (REQS; Watanabe and Riddle 2017). Exercise studies using the TreadWheel (Mendez *et al.* 2016) showed significant increases in fly protein content, which could be attributed to changes in protein metabolism (such as catabolism or synthesis). A recent GWAS study of the *Drosophila melanogaster* Genetic Reference Panel (DGRP) (Mackay *et al.* 2012) using the REQS method identified 81 variants in 47 genes associated with exercised-induced activity (Watanabe *et al*. 2020). These genes were significantly enriched for gene ontology categories relating to neuronal function (*Prosap, Spec2, nwk, Naam*, and *Wnt4*) and protein processes (*CG30463, Sh, CG33144, Ptp61F, sda*, and *CG6512*). In our study, transcriptional responses to exercise induced up-regulation of genes involved in immune-response and proteolysis, an indication that exercise may be causing inflammation and muscle damage thereby eliciting a recovery response. The TreadWheel and REQS study together with this study reveal complex responses to exercise that are affected by sex and genotype that require further exploration.

In summary, we used RNAseq and bioinformatics tools to determine which processes are mis-regulated in the presence of mutant *MYBPC3* alleles associated with feline HCM. Transcriptome analysis revealed significant downregulation of snoRNA genes in exercised female flies harboring the mutant alleles compared to flies that harbor the WT allele. Other processes that were affected included the unfolded protein response and immune/defense responses. These data suggest that mutant *MYBPC3* alters transcriptional responses of genes that could be used as targets for therapeutic interventions. Transcriptionally differentially expressed genes are also candidate genes for future evaluation as genetic modifiers of HCM as well as candidate genes for genotype by exercise environment interaction effects on the manifestation of HCM, in cats as well as humans.
